# GP100 expression is variable in intensity in melanoma

**DOI:** 10.1007/s00262-024-03776-5

**Published:** 2024-08-06

**Authors:** Jacqueline E. Mann, Nitzan Hasson, David G. Su, Adebowale J. Adeniran, Keiran S. M. Smalley, Dijana Djureinovic, Lucia B. Jilaveanu, David A. Schoenfeld, Harriet M. Kluger

**Affiliations:** 1 Division of Medical Oncology, Yale University School of Medicine, New Haven, CT USA; 2 Division of Surgical Oncology, Yale University School of Medicine, New Haven, CT USA; 3 Department of Pathology, Yale University School of Medicine, New Haven, CT USA; 4 Department of Tumor Microenvironment and Metastasis, H. Lee Moffitt Cancer Center & Research Institute, Tampa, FL USA

**Keywords:** Glycoprotein 100, ImmTAC, Melanoma, Targeted therapy, Tebentafusp

## Abstract

Drugs or cellular products that bind to gp100 are being investigated for treatment of cutaneous melanoma. The relative specificity of gp100 expression in melanocytes makes it an attractive target to harness for therapeutic intent. For example, Tebentafusp, a bispecific gp100 peptide-HLA-directed CD3 T cell engager, has generated significant enthusiasm in recent years due to its success in improving outcomes for uveal melanoma and is being studied in cutaneous melanoma. However, the extent and intensity of gp100 expression in advanced cutaneous melanoma has not been well studied. Here, we interrogated a large cohort of primary and metastatic melanomas for gp100 expression by immunohistochemistry. Expression in metastatic samples was globally higher and almost uniformly positive, however the degree of intensity was variable. Using a quantitative immunofluorescence method, we confirmed the variability in expression. As gp100-binding drugs are assessed in clinical trials, the association between activity of the drugs and the level of gp100 expression should be studied in order to potentially improve patient selection.

**Supplementary Information:**

The online version contains supplementary material available at 10.1007/s00262-024-03776-5.

## Introduction

While immune checkpoint inhibitors (ICIs) have dramatically improved treatment for advanced melanoma, responses vary across disease subtypes. Uveal melanoma accounts for 3–5% of melanomas and carries a worse prognosis, with median overall survival of less than one year in metastatic disease [1–3]. Further, while ICI regimens have substantially improved outcomes in metastatic cutaneous melanoma, they are less effective in metastatic uveal melanoma, which exhibits distinct molecular characteristics including lower mutational burden and lower PD-L1 expression [4, 5]. This disparity has spurred an ongoing effort to explore novel strategies tailored for uveal melanoma. The glycoprotein 100 (gp100) is reported to be expressed in 63–90% of melanomas [6, 7] and has long been of interest as a potential therapeutic target, leading to numerous targeted therapy trials including gp100-targeted vaccines and adoptive cell transfer. Despite occasional promising results in small-scale initial trials, gp100 targeting therapies have historically not yielded survival benefit in larger randomized trials [8–10]. More recently, a first-in-class immune-mobilizing monoclonal T-cell receptor against cancer (ImmTAC), Tebentafusp, has been developed [10]. Tebentafusp is a bispecific fusion protein comprised of an HLA-A*02:01- restricted T cell receptor that recognizes gp100 and an anti-CD3 single chain variable fragment, allowing recruitment and activation of T cells upon binding to gp100-expressing target cells [11]. A randomized trial investigated tebentafusp in uveal melanomas and demonstrated a significant improvement in overall survival compared to a control group receiving investigator’s choice of single-agent pembrolizumab, ipilimumab, or dacarbazine (21.6 months in the tebentafusp group vs 16.9 months in the control group) [11]. This result led to the 2022 approval of tebentafusp by the Food and Drug Administration for treatment of metastatic uveal melanoma in HLA-A*02:01-positive patients [12].

Given that five-year overall survival rates are > 52% with ICI in half of cutaneous melanoma patients [13–15], expanding trials of tebentafusp to the cutaneous melanoma setting is of great interest. Tebentefusp is currently being studied in a randomized trial with or without pembrolizumab in patients with non-uveal melanoma (NCT05549297). Therefore, a more nuanced understanding of gp100 expression levels and their impact on treatment response might enable clinicians to improve response rates by preferentially treating those more likely to respond. As an initial step toward this goal, the present study directly compares gp100 expression across a panel of cutaneous and uveal melanoma cell lines and interrogates the range of gp100 expression in cutaneous melanoma specimens and nevi.

## Methods

### Human cell lines

Yale University (YU)-designated cell strains were derived from human tumors as described [16]. Ten low-passage (< 20) melanoma cultures were obtained from the Biospecimen Core of the Yale SPORE in Skin Cancer (Table S1). All YU-designated cell lines were maintained in OptiMEM media (Invitrogen) supplemented with 10% heat-inactivated FBS (Invitrogen) and 1% antibiotic-antimycotic (penicillin, streptomycin, amphotericin B). Uveal melanoma cell lines 92.5, OMM1, MP38, Mel202, Mel270, Mel290, and MP41 were provided by Dr. Keiran Smalley and maintained in RPMI supplemented with 10% FBS, 1% Penicillin-Streptomycin, and 1% Non-essential amino acids. Cells were incubated at 37 °C in a humidified atmosphere of 95% air/5% CO2. Mutation status for BRAF and NRAS was retrieved from the DepMap portal (https://depmap.org/portal) for non-YU uveal melanoma cell lines.

### Western blotting

Western blotting was performed by standard methods. For gp100 detection we used monoclonal rabbit anti-human gp100 (Novus) diluted at 1:1000. Quantitation was done using Image J.

### Patient cohort and tissue microarray construction

The melanoma and nevi tissue microarrays were constructed as previously described [17, 18]. Collection of patient specimens and clinical data was approved by the Yale University Institutional Review Board. Representative regions of invasive tumor were examined by a pathologist and a 0.6 mm diameter core was obtained from the tumor region for each specimen. Cores from 230 primary melanomas and 293 metastatic melanomas resected between 1959 and 2000 were arranged in a tissue microarray. Of these, 119 primary tumors and 209 metastases had sufficient viable tumor tissue for analysis. Patients were 55% male with age of diagnosis ranging from 18 to 91 years (mean = 52.4 years), and follow-up ranged from 2 months to 40 years (mean = 6.7 years). Anatomic sites of metastatic lesions included lymph node (48%), skin (32%), abdominal viscera (7.2%), bone (3.0%), and lung (3.0%). Other sites (breast, brain, nasal/oral mucosa, muscle, pericardium, and pleura) were each represented by < 1% of metastatic cores. BRAF^V600E^ staining was positive in 64% of cores [19]. The nevus array contains cores from 263 benign lesions as well as 40 metastatic or primary specimens also represented on the melanoma array. Both arrays contained identical cell lines, cored from pellets. Overlapping metastatic and primary specimens and cell lines were used for normalization of the scores obtained from the benign and malignant arrays.

### Immunohistochemistry

For immunohistochemistry (IHC), 5 µm TMA sections were mounted on glass slides, incubated with 0.5% bovine serum albumin in tris buffered saline for 30 min, and incubated at 4 °C overnight slides with the antibody HMB45 (BioGenex diluted 1:250). Slides were incubated with horseradish peroxidase-conjugated anti-rabbit secondary antibody (Envision) for 2 h at room temperature and stained with 3-Amino-9-Ethylcarbazole (AEC) substrate solution (Abcam ab64252; red stain) and hematoxylin per manufacturer’s instructions. Immunohistochemistry staining was evaluated by an independent pathologist (A.A) blinded to the clinical data. Tissue staining was largely uniform within the histospots and were therefore scored on a scale of 0–3 for negative, weak, moderate and strong staining.

### Immunofluorescence

Immunofluorescence staining was performed as previously described [17]. Slides were incubated with the HMB45 antibody (BioGenex) diluted at 1:250 at 4 °C overnight. Anti-rabbit horseradish peroxidase-conjugated secondary antibody (Envision) was applied for signal amplification and Cy5-tyramide (NED Life Science Products) was used for visualization of gp100. To distinguish tumor from stroma, slides were incubated with mouse anti-S100 at 1:100 (ThermoFisher, clone 15E2E2). Cy3-tyramide (NED Life Science Products) was used for visualization of S100. Slides were incubated with 4,6-diamidine-2-phenylin- dole (DAPI, 1:200, Invitrogen, Carlsbad, CA) for nuclear staining.

Monochromatic images were obtained and analyzed using algorithms previously described [20, 21]. Briefly, DAPI staining was used to generate a total tissue mask. The S100 (Cy3) signal was used to generate a tumor mask. A rapid exponential subtraction algorithm was used to subtract out-of-focus information and each pixel was assigned to a tumor or stroma compartment. Gp100 (Cy5) signal intensity was calculated as an average signal of the pixels comprising the tumor compartment. Histospots containing insufficient tissue (< 3% of the core area) or abundant necrotic tissue were excluded. Linear regression was performed to assess correlation between control specimens represented on both arrays, and the resulting equation was used to compute adjusted scores for the melanoma array.

## Results

### *gp100 expression in melanoma cell lines*

**Fig. 1:**
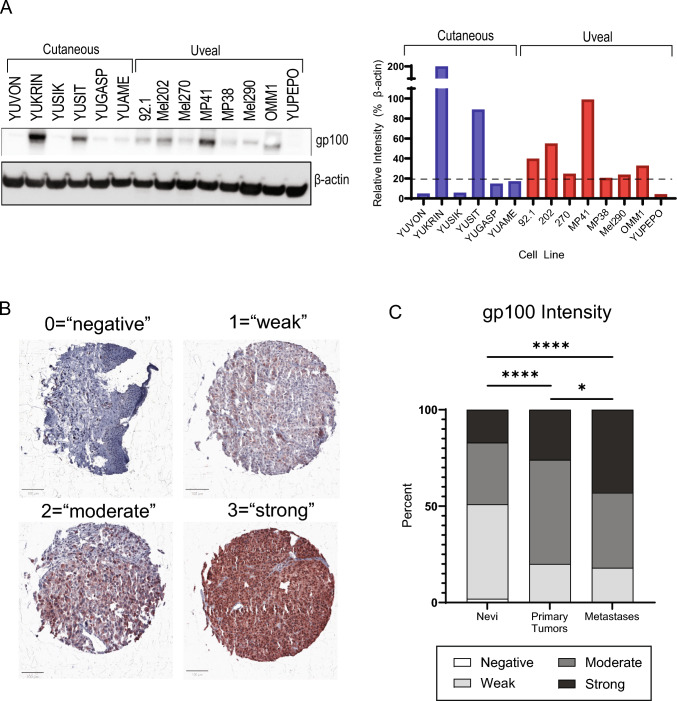
Detection of gp100 protein in melanoma cell lines and tissues. **A** Western blot showing expression of gp100 (100kD) in cell lines derived from cutaneous and uveal melanoma cell lines (left). Pixel intensity was quantitated for each band using ImageJ and displayed relative to loading control (right). “Strong expression” was defined as relative intensity > 20 and indicated by dashed line. Blue, cutaneous melanoma-derived; red, uveal melanoma-derived. **B** Representative images from immunohistochemical staining of the nevus and melanoma tissue microarrays showing scores from 0 to 3. Top left, nevus; top right, primary melanoma, lower left, metastatic melanoma; lower right, metastatic melanoma. **C** Intensity scores for nevus and melanoma tissue microarrays. *p < 0.01, ****p < 0.0001

We first compared expression of gp100 in a panel of 8 uveal and 10 cutaneous melanoma cell lines by Western blot and calculated band intensity relative to loading control. Strong gp100 expression (relative intensity > 20) was observed in 7/8 (88%) uveal melanoma cell lines and 4/10 (40%) cutaneous melanoma cell lines (Fig. 1A, S1). The highest gp100 expression was observed in the cutaneous line YUKRIN. Although the small sample size prevented robust statistical analysis, mean gp100 expression was similar in cutaneous and uveal melanoma, yet expression levels were more variable in cutaneous lines, which exhibited a mean relative intensity of 41.3 and standard deviation of 58.8. Expression in uveal melanoma exhibited a mean relative intensity of 37.3 and standard deviation of 27.1. Thus, it appears that uveal melanoma cell lines express more consistently high levels of gp100, while cutaneous lines represent a diverse range.

### gp100 expression in human tumors by immunohistochemistry

We performed IHC in two tissue microarrays (TMAs), one consisting of 119 primary melanomas and 210 metastases, and a second consisting of 252 nevi. Figure 1B shows examples of positive and negative gp100 immunoreactivity in representative cores. Less than 50% of benign nevi exhibited moderate or strong gp100 expression, whereas > 80% of melanomas were either moderately or strongly positive (Fig. 1C). Strong expression was most prevalent in metastases (43% vs 26% in primary tumors).

### gp100 expression and associations with clinical characteristics

The melanomas were annotated for clinical characteristics. No clear association was found within either the primary or metastatic cohorts between the degree of gp100 immunoreactivity and overall survival. Similarly, no association was found between gp100 intensity and clinical or pathological characteristics (depth, ulceration, mitoses, gender or age among primary melanomas and BRAF status, gender or age within the metastatic disease cohort).

### Quantitative immunofluorescence measures of gp100 expression

**Fig. 2:**
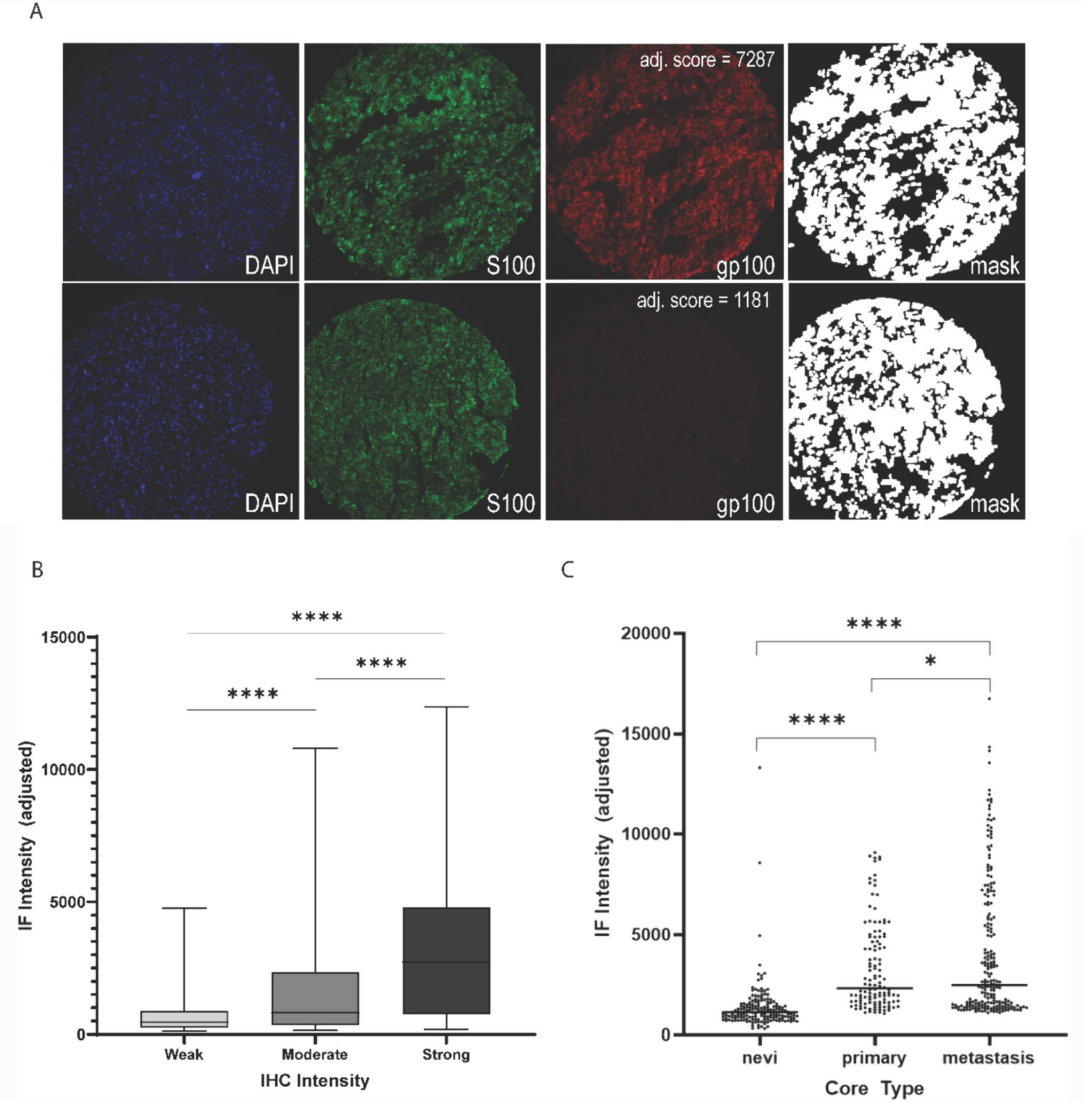
Immunofluorescence staining of gp100 in melanoma tissues. **A** Representative pseudo-color images for two metastatic melanoma histospots with high (top) and low (bottom) IF intensity scores. Staining target is indicated in lower right for each image. Tumor masks generated from analysis of S100 staining are shown for each histospot, and IF intensity score is determined for gp100 in the tumor compartment. **B** Box plot showing IF scores from histospots that received both a QIF score and an IHC score (Fig. 1). Histospots were grouped by IHC score as indicated. ****p < 0.0001. **C** Box plot showing IF scores for nevi, primary melanomas, and metastatic melanomas. *p < 0.01, ****p < 0.0001

Given that the naked eye cannot distinguish small differences in intensity, we used quantitative immunofluorescence (QIF) to better appreciate the range of gp100 expression in cutaneous melanoma. This method correlates well with expression as measured by other methods [20]. QIF staining is depicted in Fig. 2A. The two TMAs contained matching histospots from the same tumor. QIF scores were highly correlated in matching spots (r = 0.9, Fig. S2), although staining on the melanoma array was somewhat weaker, likely due to age of the section. Scores for the melanoma array were therefore normalized to the nevus array. QIF scores ranged from 1000 to 17,000. Importantly, QIF scores correlated well with the IHC scores (*p* < 0.0001), but within each IHC group (weak, moderate or strong staining) there was substantial variability (Fig. 2B). As with the IHC, gp100 expression as quantified by QIF in melanomas was significantly higher than nevi and higher in metastases than primary lesions (*p* < 0.0001 and *p* = 0.03, respectively; Fig. 2C). As gp100-targeting therapeutics are being investigated for patients with metastatic cutaneous melanoma, we note that while these specimens almost universally expressed gp100, expression levels were quite variable (Fig. 2C).

## Discussion

Gp100 is emerging as an important target in melanoma given its specificity for melanocytes. Tebentafusp represents a major advancement in the treatment of uveal melanoma, and initial trials in cutaneous melanoma have yielded promising results [13]. Along with ongoing phase 3 studies of tebentafusp, other gp100-targeted treatment modalities are being studied for melanoma patients, including alternative bi-specific antibodies and cell therapy approaches. However, an association between gp100 levels and response to these treatments has not been established. We report moderate or strong gp100 expression in over 80% of cutaneous melanomas in our cohort, supporting further investigation of gp100 targeted agents in this population. Further, we observed variability in the degree of gp100 expression in both primary and metastatic melanomas, provoking consideration of whether patient stratification according to gp100 expression could refine the target population for these treatments and improve response rates. The findings herein expand on previous reports describing heterogeneous gp100 expression and correlation with disease stage by utilizing a larger cohort and quantitative methods revealing a broad range of expression even among metastatic lesions.

Numerous gp100 targeted adoptive cell therapies [22] and vaccines [10, 11] have been investigated in clinical trials, and these strategies continue to be explored using more contemporary strategies. The peptide vaccine Gp-100:209-217 improved the response rate of interleukin-2 in a Phase III trial, but did not yield a statistically significant survival benefit and is therefore not approved [23]. Cellular therapies have also been designed to target gp100. For example, a small phase II trial of TCR engineered lymphocytes targeting gp100 was conducted (NCT00509496) and responses were observed in a subset of patients [24]. Oncolytic viruses binding gp100 positive cells have also been studied (NCT01008527). A role for gp100 expression in therapeutic response has not been established, but it is possible that lack of baseline expression or loss of expression of gp100 with time may emerge as a mechanism of resistance to gp100 targeting therapies [25]. Importantly, while the present study describes variability in gp100 expression between patients, cores may not represent the entire specimen and we are therefore unable to assess intratumoral heterogeneity. We note that in clinical practice, biomarker analysis for metastatic patients is typically done using core biopsies of similar diameter to tissue microarray cores. Additional studies are needed on whole tumor resection specimens to determine whether there is heterogenous expression within a tumor. Such intratumoral heterogeneity may suggest mechanisms of treatment resistance, such as outgrowth of low antigen expressing tumor cells.

While the significance of gp100 expression levels in predicting response to gp100 targeted agents remains to be investigated, correlation of target expression with therapeutic response is well documented in other settings. For example, numerous studies in HER2 + breast cancer patients have demonstrated differential response to anti-Her2/Neu antibodies based on target expression levels [26]. Tebentafusp represents an attractive strategy for managing subsets of cutaneous melanomas that have progressed on ICI regimens. As ongoing trials evaluate tebentafusp in cutaneous melanoma patients following disease progression on anti-PD1 therapy (NCT05549297), it will be critical to determine whether there is a threshold for gp100 expression below which patients are unlikely to respond. This will enable clinicians to direct these patients to alternative therapies. Further, eligibility for tebentafusp is HLA-restricted, and as similar agents are developed using less common HLA types, insights into patient stratification gained from tebentafusp studies will be valuable.

Although tebentafusp is the first FDA-approved gp100 targeted therapy, new molecular and cellular technologies are emerging and tumor-specific targets to which antibodies and cells can bind, such as gp100, are important for minimizing off-target effects. Here we show that while some expression of gp100 is seen in most metastatic cutaneous melanomas, the degree of expression is variable. It is possible that responses are only seen when the target is expressed at higher levels, and our results suggest that quantitative measures of gp100 should be incorporated into clinical trials with gp100 targeting antibodies or cellular products.

## Supplementary Information

Below is the link to the electronic supplementary material.

Supplementary file 1. Fig. S1 Western blot analysis of additional cutaneous melanoma cell lines. Fig. S2 Correlation of IF scores for matched histospots on nevus and melanoma TMAs. (PDF 582 kb)

Supplementary file 2. (XLSX 10 kb)

## Data Availability

No datasets were generated or analysed during the current study.
