# Supplementary figures and images for: GP100 expression is variable in intensity in melanoma
Source: Cancer Immunol Immunother. 2024 Aug 6;73(10):191. doi: 10.1007/s00262-024-03776-5 (PMC11303354; doi:10.1007/s00262-024-03776-5)

Figure S1

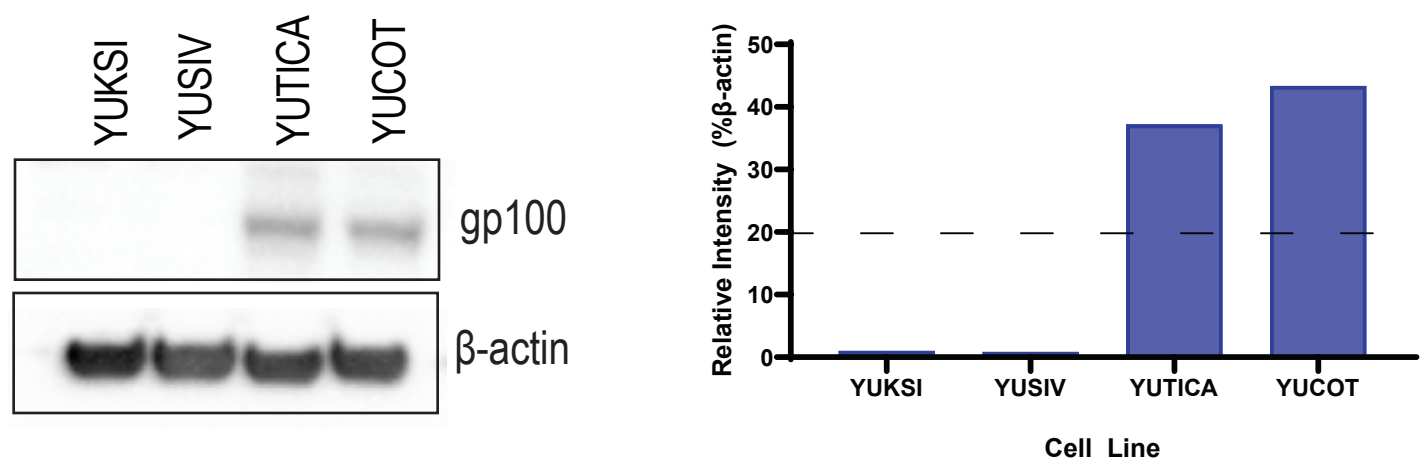

Figure S2

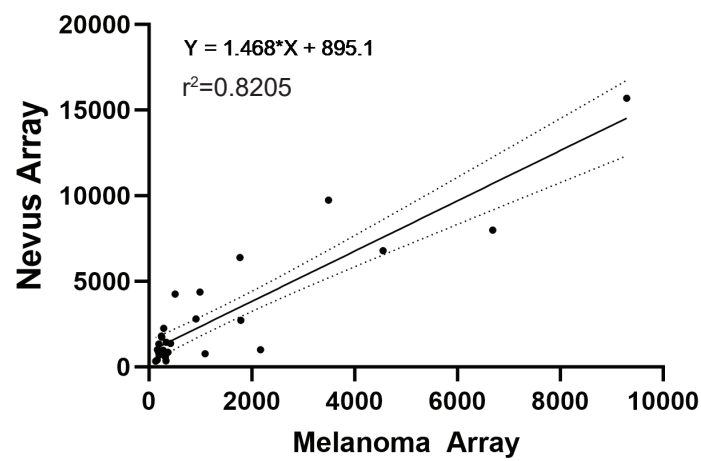

Supplement: Supplementary file 1 — Fig. S1 Western blot analysis of additional cutaneous melanoma cell lines. Fig. S2 Correlation of IF scores for matched histospots on nevus and melanoma TMAs. (PDF 582 kb) [file 262_2024_3776_MOESM1_ESM.pdf]
